# Supplementary material for: Interplay between Optical Emission and Magnetism in the van der Waals Magnetic Semiconductor CrSBr in the Two-Dimensional Limit
Source: ACS Nano. 2023 Jul 13;17(14):13224–31. doi: 10.1021/acsnano.3c00375 (PMC10863932; doi:10.1021/acsnano.3c00375)
Supplement: Supplementary file 1 — nn3c00375_si_001.pdf [file nn3c00375_si_001.pdf]

Supporting information

Interplay between optical emission and magnetism in the van der Waals magnetic semiconductor CrSBr in the two-dimensional limit.

Francisco Marques-Moros, Carla Boix-Constant, Samuel Mañas-Valero, Josep Canet-Ferrer, and Eugenio Coronado**

Instituto de Ciencia Molecular (ICMol), Universitat de València, Paterna, Spain.

Corresponding Authors: eugenio.coronado@uv.es, jose.canet-ferrer@uv.es

Supporting Information SI1: Temperature dependence of the PL emission for CrSBr for different thicknesses

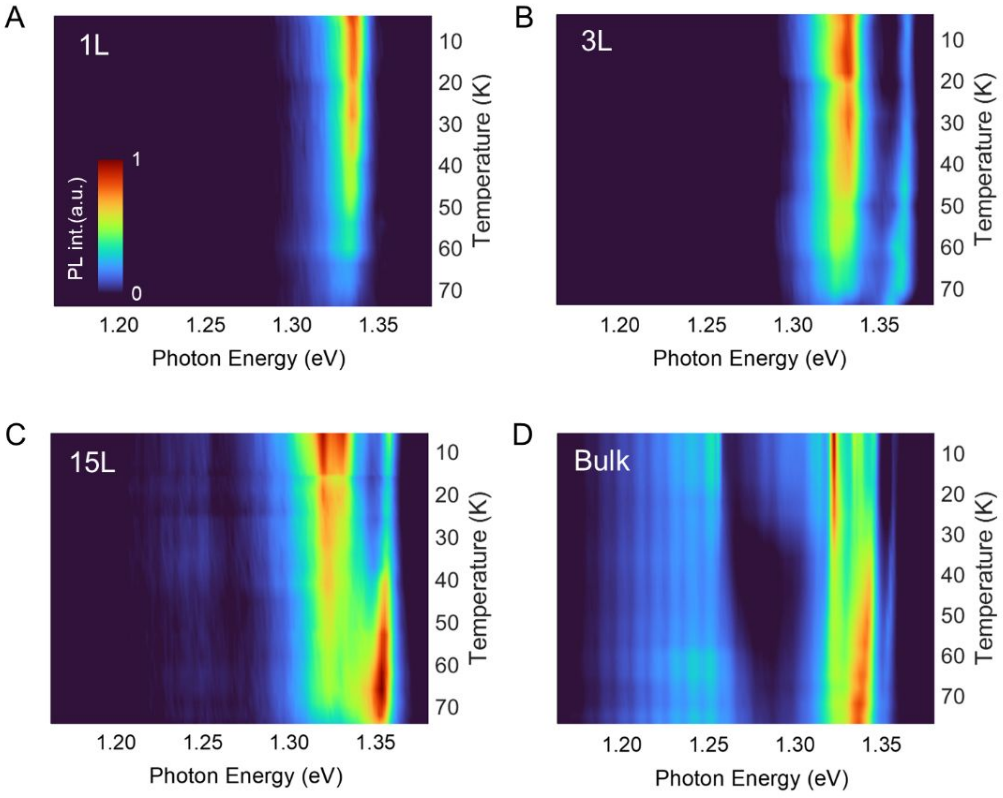

Figure SI1. PL integrated area dependence on the temperature in the range of the hidden order for a monolayer (A), trilayer (B), multilayer (15L) (C), and bulk-like flake (D).

Supporting Information SI2: Analysis of the Temperature dependent PL measurements.

In order to distinguish the different carrier transfer mechanisms in CrSBr and extract information about its exciton recombination, we estimated the activation energies of every mechanism. To do so, we fit the experimental data of PL intensity (extracted from the PL band Lorentzian deconvolution) as a function 1/T. The empirical formula universally used to fit the PL intensity dependence is at follows:

$$I(T) = \frac{I(0)}{1 + \left[G_i \exp\left(-\frac{E_i}{kT}\right)\right]} \quad (1)$$

where I(0) is the optical intensity at 0K (experimentally we take the lowest temperature value in our measurements, 4 K), G_i the scattering rate, E_i the activation energy of loss mechanism, and *k* is the Boltzmann's constant.[1,2]

Generally, any PL quenching mechanism (namely the ith mechanism) can be expressed by an Arrhenius-type equation, which then is used to calculate corresponding the activation energy E_i and rate G_i. In this way, the activation energies are obtained from the slopes of those plots. This method has been extensively used in literature for this purpose.[3–5] One of its advantages is that it is less sensitive broadening distribution of the PL, so it can be used at higher temperatures, if compared with the Arrhenius plots of the PL decay times.[4]

Occasionally, a single thermal activation energy is not enough to fit the Arrhenius plot from the experimental temperature-dependent integrated PL intensity. This fact suggests that there are more loss mechanisms involved in the PL quenching as the temperature increases (see Eq. 1).[6] In addition, apart from the usual quenching of the PL with the temperature, some systems display a PL increase due to a carrier injection from another mechanism in a certain range of temperatures.[7,8] In this way, it is necessary to incorporate a feeding mechanism in the expression in order to fit the experimental data (see Eq. 2).

In this way, in the temperature ramp described in the main text, we have applied different expressions to each emission peak. Hence, the following equations incorporate different activation energies, with different quenching and feeding mechanisms. These activation energies will be related together for explaining our system dynamics and compared with values published in the literature for different possible quenching and feeding mechanisms:

First, P1 presents a typical behavior with two non-radiative mechanisms and fits with the following expression containing two Boltzmann-type quenching mechanisms:[3,8–10]

$$I(T) = \frac{I(0)}{1 + \left[G_1 \exp\left(-\frac{E_1}{kT}\right) + G_2 \exp\left(-\frac{E_2}{kT}\right)\right]} \qquad (2)$$

As mentioned in the main text, the low activation energies observed in this peak [E_1(P1) = 1.2 meV and E_2(P1) = 14 meV] are typically ascribed to non-radiative mechanism related with defects or impurities.[11,12] We should expect a similar behavior from P3 given the similar temperature dependence, however, the best fitting for the case of P3 is obtained with a single non-radiative mechanism with an activation energy in between E_1(P1) and E_2(P1) [E_1(P3) = 4.1 meV].

On the other hand, the emission of P2 quenches in favor of P4 at low temperatures. However, above 100 K this trend is reversed and the contribution of P2 to the PL is enhanced again, as concluded from the peak deconvolution. This PL enhancement at higher temperatures is represented by a carrier feeding mechanism, between brackets in Eq. 3. This feeding factor considers the ratio between the carrier injection and the radiative recombination rate of P2:[7,8]

$$I(T) = \frac{I(0)}{1 + \tau_d \left[G_1 \exp\left(-\frac{E_1}{kT}\right) + G_2 \exp\left(-\frac{E_4}{kT}\right) \right]} * \left[1 + \frac{A_3}{1 + G_3 \exp\left(\frac{E_3}{kT}\right)} \right] \quad (3)$$

Here, A is the carrier population ratio being transferred to the flake (probably from other peaks). As discussed in the main text, the activation energy of this mechanism (E_3) is similar to quenching mechanism of P4 [E_3(P4)]. For this reason, the quenching of P2 around 20 K would be associated with E1 (also observed in P1 and P3) while the

contribution of E_2(P1) would be hindered by the strong influence of the feeding mechanism in the fitting, E_3(P2) = 35 meV. Then, G_3 would be the ratio between the radiative recombination rate of P2 the injection rate from P4. Lastly, there is an additional loss mechanism that occurs at higher energies, E_4(P2) = 72 meV. We can explain this mechanism by the unipolar escape of electrons, even the value of E_4(P2) is high if compared with this phenomenon in quantum wells.[11]

Finally, the temperature evolution of P4 (closely related to P2) fits to the next expression with one quenching mechanism and one feeding mechanism:[8]

$$I(T) = \frac{I(0)}{1 + \tau_d G_3 \exp\left(-\frac{E_3}{kT}\right)} * \left[1 + \frac{A}{1 + G_5 \exp\left(\frac{E_5}{kT}\right)}\right] \quad (3)$$

Simultaneously to the quenching of P2, there is an increase in the PL intensity of P4. This feeding mechanism (E_5(P4) = 3.7 meV) is correlated with the ionization of donor impurities [E_1(P2) = 1.5 meV] and will drive to an increase of uncorrelated electrons in the conduction band. Later, P4 quenches in favor of P2 by a mechanism with an activation energy E_3(P4) = 39 meV. It is worth mentioning that the enhancement or the quenching of those peaks will differ from the typical charge transfer observed in semiconductor nanostructures,[14–18] as described in the main text.

Supporting Information SI3: Lorentzian deconvolution of the multilayer PL spectra at relevant temperatures

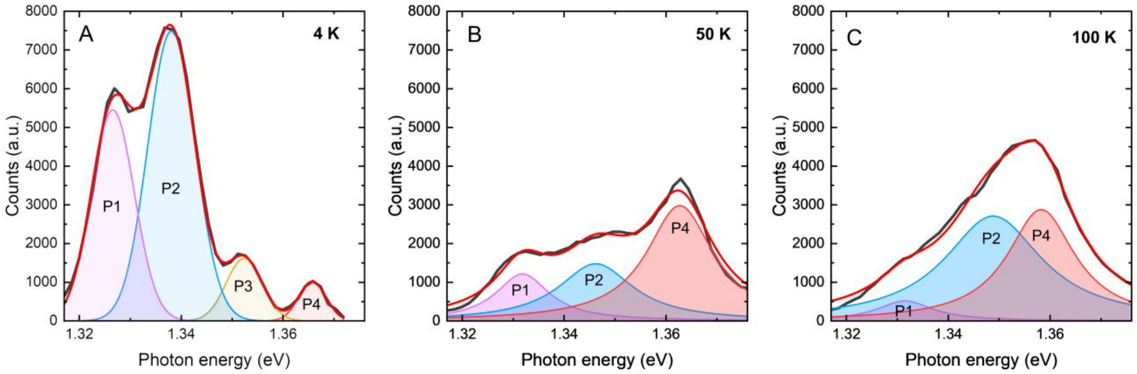

Figure SI2. B2 PL Band Gaussian deconvolution for 3 representative temperatures for a multi-layer CrSBr flake. (A) 4 K deconvolution with P1 and P2 dominating the spectrum. (B) 50 K band deconvolution after the interplay between P1-P2 and P4, with P3 quenching. (C) shows the 100 K deconvolution, where P2 starts becoming dominant all over the PL signal.

Supporting Information SI4: Differential Reflectivity measurements on few-layer and multilayer CrSBr flakes

Differential reflectivity measurements have been carried out by using a tungsten lamp coupled to a multimode fiber in confocal conditions. We measured two different few-layered flakes with different thicknesses, a 15- and a 6- layer flake, at 10K. As reported for previous authors,[19] the reflectivity signal is expected to shift approximately 15 meV respect to the PL spectrum. In our case we observe a similar result with a shift of 15meV in our flakes. The results are shown in Figure SI3.

In this way, the peak P4 (emitting at 1.36 eV), appears as a little dip at 1.375 eV in the differential reflectivity spectra. This feature is very weak if compared with the contribution of the peaks P1-P2, pointing that the oscillator strength of P4 is smaller,[20,21] as expected for multielectron transitions.[22–24]

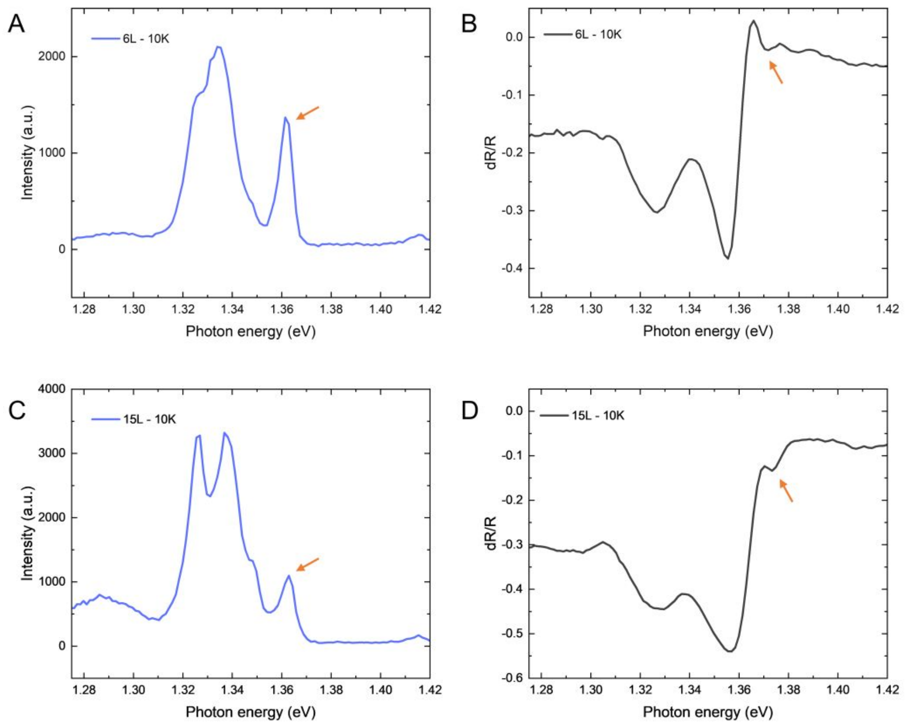

Figure SI3. (A) and (C) show the PL spectra of a 6- and a 15-layer CrSBr flakes at 10K, respectively. (B) and (D) show the corresponding differential reflectivity. Orange arrows point at the higher energy peak P4.

Supporting Information SI5: Excitation power dependence of the emission in multilayer CrSBr flakes

The photoluminescence dependence on the excitation power of a 6-layer CrSBr flake at 10 K is shown in Fig. SI4. A CW laser diode operating at 530 nm wavelength is used as excitation source. The integrated intensity of P2 and P4 are shown in Fig. SI4A. This is estimated from the Lorentzian deconvolution, see some representative examples for high

and low powers in Figs. SI4B and SI4C, respectively. Unfortunately, we cannot reach the saturation regime with our current set-up configuration.

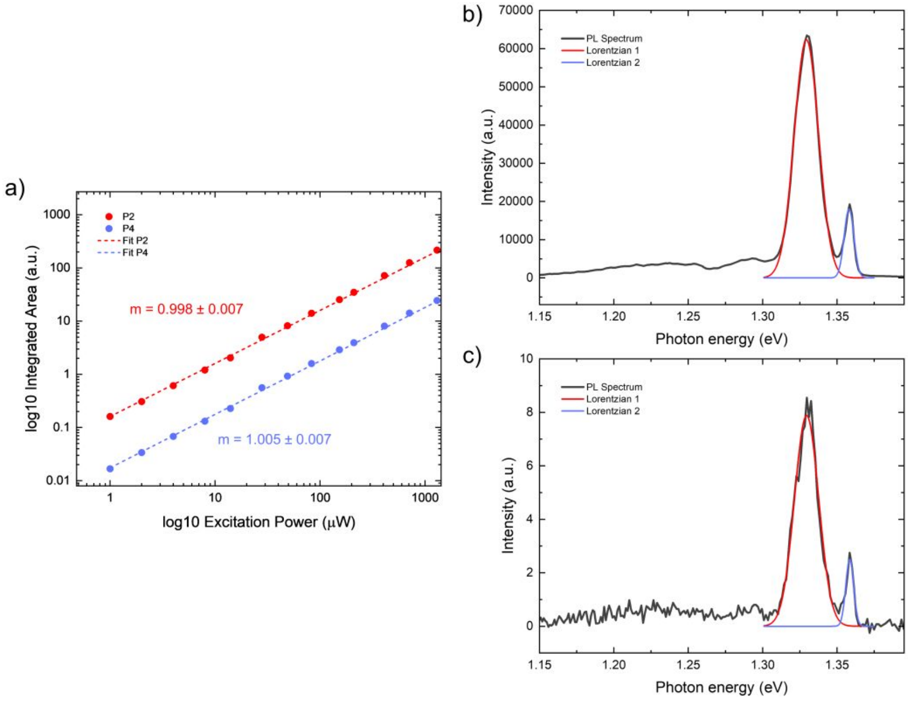

Figure SI4. (A) Double logarithmic plot of the integrated PL area of P2 and P4 for a 6-layer CrSBr flake at 10K. In both cases, we can estimate a slope near-to-one, as labeled in the plot. (B) and (C) show two representative spectra at high and low power, respectively. The corresponding Lorentzian peak deconvolution is plotted together with the spectra in red for P2 and blue for P4.

Supporting Information SI5: Time resolved photoluminescence of the emission in multilayer CrSBr flakes

Time resolved measurements have been carried out in a range of CrSBr layers with different thickness. A picosecond pulsed diode laser operating at 810 nm wavelength with

a repetition rate of 40 MHz has been used as excitation source. The PL excitation is filtered by means of a couple of edge filters. The filtered signal is analyzed using a two-exit monochromator provided with back thinned Si-CCD camera and a Single Photon Avalanche Diode (SPAD). The transients are acquired through a time-tagger (Pico Harp 300) and analyzed with the corresponding software. In all the flakes analyzed the decay times are very fast, sometimes within the experimental error (estimated around 50 ps after the deconvolution of the system response). As an example, in Fig. SI5, we plot the transients obtained for P4 (40 ± 50 ps) and P2 (80 ± 50 ps).

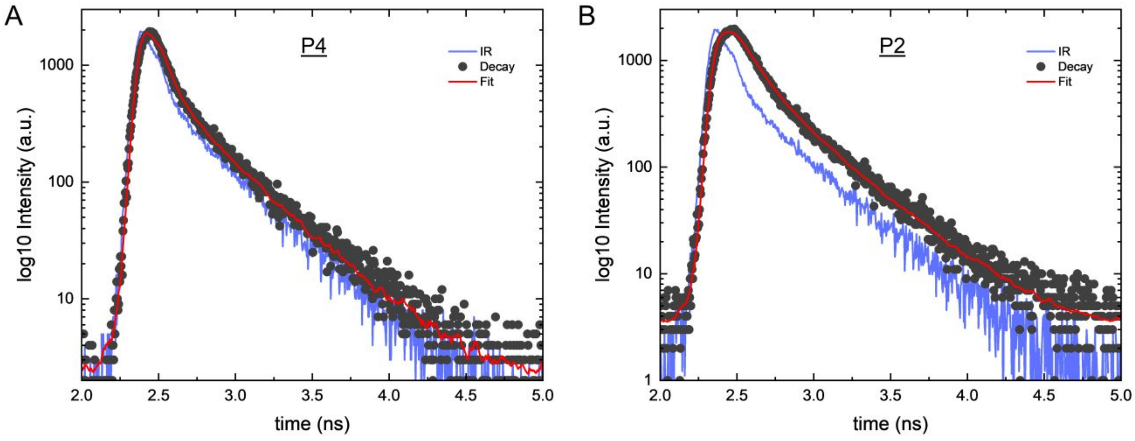

Figure SI5. (A) and (B) PL decays for the 15-layer CrSBr emission at 60 K filtered at 905 nm and 920 nm, which correspond to P4 and P2, respectively.

REFERENCES

(1) Shionoya, S. Photoluminescence. In *Luminescence of Solids*; Vij, D. R., Ed.; Springer US: Boston, MA, 1998; pp 95–133. https://doi.org/10.1007/978-1-4615-5361-8_3.

(2) Hao, M.; Zhang, J.; Zhang, X. H.; Chua, S. Photoluminescence Studies on InGaN/GaN Multiple Quantum Wells with Different Degree of Localization. *Appl Phys Lett* **2002**, *81* (27), 5129–5131. https://doi.org/10.1063/1.1531837.

(3) Bimberg, D.; Sondergeld~, M.; Grobe, E. *Thermal Dissociation of Excitons Bounds to Neutral Acceptors in High-Purity GaAsg*; Vol. 4.

(4) Chen, Y.; Kiba, T.; Takayama, J.; Higo, A.; Tanikawa, T.; Chen, S.; Samukawa, S.; Murayama, A. Temperature-Dependent Radiative and Non-Radiative Dynamics of Photo-Excited Carriers in Extremely High-Density and Small InGaN Nanodisks Fabricated by Neutral-Beam Etching Using Bio-Nano-Templates. *J Appl Phys* **2018**, *123* (20). https://doi.org/10.1063/1.5027627.

(5) Leroux, M.; Grandjean, N.; Beaumont, B.; Nataf, G.; Semond, F.; Massies, J.; Gibart, P. Temperature Quenching of Photoluminescence Intensities in Undoped and Doped GaN. *J Appl Phys* **1999**, *86* (7), 3721–3728. https://doi.org/10.1063/1.371242.

(6) Hugues, M.; Damilano, B.; Duboz, J. Y.; Massies, J. Exciton Dissociation and Hole Escape in the Thermal Photoluminescence Quenching of (Ga,In)(N,As) Quantum Wells. *Phys Rev B Condens Matter Mater Phys* **2007**, *75* (11). https://doi.org/10.1103/PhysRevB.75.115337.

(7) Ouerghui, W.; Martinez-Pastor, J.; Gomis, J.; Melliti, A.; Maaref, M. A.; Granados, D.; Garcia, J. M. Effect of Carrier Transfer on the PL Intensity in Self-Assembled in (Ga) As/GaAs Quantum Rings. *EPJ Applied Physics* **2006**, *35* (3), 159–163. https://doi.org/10.1051/epjap:2006088.

(8) Canet-Ferrer, J.; Munoz-Matutano, G.; Fuster, D.; Alen, B.; Gonzalez, Y.; Gonzalez, L.; Martinez-Pastor, J. P. Localization Effects on Recombination Dynamics in InAs/InP Self-Assembled Quantum Wires Emitting at 1.5 m. In *Journal of Applied Physics*; 2011; Vol. 110. https://doi.org/10.1063/1.3660260.

(9) Adelmann, C.; Simon,) J; Feuillet, G.; Pelekanos, N. T.; Daudin, B.; Fishman, G. *Self-Assembled InGaN Quantum Dots Grown by Molecular-Beam Epitaxy*; 2000; Vol. 76. http://ojps.aip.org/aplo/aplcr.jsp.

(10) Luckert, F.; Yakushev, M. V.; Faugeras, C.; Karotki, A. V.; Mudryi, A. V.; Martin, R. W. Excitation Power and Temperature Dependence of Excitons in CuInSe 2. *J Appl Phys* **2012**, *111* (9). https://doi.org/10.1063/1.4709448.

(11) Cunningham, J. E.; Timp, G.; Chang, A. M.; Chiu, T. H.; Jan, W.; Schubert, E. F.; Tsang, W. T. *SPATIAL LOCALIZATION OF Si IN SELECTIVELY 8-DOPED Al~Ga 1~As/GaAs HETEROSTRUCTURES FOR HIGH MOBILITY AND DENSITY REALIZATION*; 1989; Vol. 95.

(12) Fuster, D.; González, L.; González, Y.; Martínez-Pastor, J.; Ben, T.; Ponce, A.; Molina, S. I. Emission Wavelength Engineering of InAs/InP(001) Quantum Wires. *European Physical Journal B* **2004**, *40* (4), 433–437. https://doi.org/10.1140/epjb/e2004-00228-4.

(13) Rivas, D.; Muñoz-Matutano, G.; Canet-Ferrer, J.; García-Calzada, R.; Trevisi, G.; Seravalli, L.; Frigeri, P.; Martínez-Pastor, J. P. Two-Color Single-Photon Emission from InAs Quantum Dots: Toward Logic Information Management Using Quantum Light. *Nano Lett* **2014**, *14* (2), 456–463. https://doi.org/10.1021/nl403364h.

(14) Kim, H.; Yoon, Y. G.; Ko, H.; Kim, S. M.; Rho, H. Charge Transfer across Monolayer/Bilayer MoS2 Lateral Interface and Its Influence on Exciton and Trion Characteristics. *2d Mater* **2019**, *6* (2). https://doi.org/10.1088/2053-1583/aafa52.

(15) Zimmermann, J. E.; Axt, M.; Mooshammer, F.; Nagler, P.; Schüller, C.; Korn, T.; Höfer, U.; Mette, G. Ultrafast Charge-Transfer Dynamics in Twisted MoS2/WSe2Heterostructures. *ACS Nano* **2021**, *15* (9), 14725–14731. https://doi.org/10.1021/acsnano.1c04549.

(16) Zhang, J.; Guan, M.; Lischner, J.; Meng, S.; Prezhdo, O. v. Coexistence of Different Charge-Transfer Mechanisms in the Hot-Carrier Dynamics of Hybrid Plasmonic Nanomaterials. *Nano Lett* **2019**, *19* (5), 3187–3193. https://doi.org/10.1021/acs.nanolett.9b00647.

(17) Zhu, H.; Yang, Y.; Wu, K.; Lian, T. Charge Transfer Dynamics from Photoexcited Semiconductor Quantum Dots. *Annu Rev Phys Chem* **2016**, *67*, 259–281. https://doi.org/10.1146/annurev-physchem-040215-112128.

(18) Muñoz-Matutano, G.; Suárez, I.; Canet-Ferrer, J.; Alén, B.; Rivas, D.; Seravalli, L.; Trevisi, G.; Frigeri, P.; Martínez-Pastor, J. Size Dependent Carrier Thermal Escape and Transfer in Bimodally Distributed Self Assembled InAs/GaAs Quantum Dots. *J Appl Phys* **2012**, *111* (12). https://doi.org/10.1063/1.4729315.

(19) Wilson, N. P.; Lee, K.; Cenker, J.; Xie, K.; Dismukes, A. H.; Telford, E. J.; Fonseca, J.; Sivakumar, S.; Dean, C.; Cao, T.; Roy, X.; Xu, X.; Zhu, X. Interlayer Electronic Coupling on Demand in a 2D Magnetic Semiconductor. *Nat Mater* **2021**, *20* (12), 1657–1662. https://doi.org/10.1038/s41563-021-01070-8.

(20) Tołłoczko, A.; Oliva, R.; Woźniak, T.; Kopaczek, J.; Scharoch, P.; Kudrawiec, R. Anisotropic Optical Properties of GeS Investigated by Optical Absorption and Photoreflectance. *Mater Adv* **2020**, *1* (6), 1886–1894. https://doi.org/10.1039/d0ma00146e.

(21) Munkhbat, B.; Baranov, D. G.; Bisht, A.; Hoque, M. A.; Karpiak, B.; Dash, S. P.; Shegai, T. Electrical Control of Hybrid Monolayer Tungsten Disulfide-Plasmonic Nanoantenna Light-Matter States at Cryogenic and Room Temperatures. *ACS Nano* **2020**, *14* (1), 1196–1206. https://doi.org/10.1021/acsnano.9b09684.

(22) Esser, A.; Zimmermann, R.; Runge, E. Theory of Trion Spectra in Semiconductor Nanostructures. *Phys Status Solidi B Basic Res* **2001**, *227* (2), 317–330. https://doi.org/10.1002/1521-3951(200110)227:2<317::AID-PSSB317>3.0.CO;2-S.

(23) Ye, J.; Yan, T.; Niu, B.; Li, Y.; Zhang, X. Nonlinear Dynamics of Trions under Strong Optical Excitation in Monolayer MoSe2. *Sci Rep* **2018**, *8* (1). https://doi.org/10.1038/s41598-018-20810-6.

(24) Ayari, S.; Quick, M. T.; Owschimikow, N.; Christodoulou, S.; Bertrand, G. H. V.; Artemyev, M.; Moreels, I.; Woggon, U.; Jaziri, S.; Achtstein, A. W. Tuning Trion Binding Energy and Oscillator Strength in a Laterally Finite 2D System: CdSe Nanoplatelets as a Model System for Trion Properties. *Nanoscale* **2020**, *12* (27), 14448–14458. https://doi.org/10.1039/d0nr03170d.
